# Pyrene-BODIPY-substituted novel water-soluble cyclotriphosphazenes: synthesis, characterization, and photophysical properties

**DOI:** 10.3906/kim-1907-40

**Published:** 2020-02-11

**Authors:** Seda ÇETİNDERE, Serkan YEŞİLOT, Adem KILIÇ

**Affiliations:** 1 Department of Chemistry, Gebze Technical University, Gebze, Kocaeli Turkey; 2 Institute of Inorganic Chemistry I, Ulm University, Ulm Germany

**Keywords:** Cyclotriphosphazene, BODIPY, pyrene, water-soluble, photophysical

## Abstract

In the present work, pyrene-boron-dipyrromethene (BODIPY)-substituted novel water-soluble cyclotriphosphazene derivatives (6 and 7) were synthesized by click reactions between a cyclotriphosphazene derivative with a hydrophilic glycol side group (2) and BODIPYs (4 and 5). All of the new compounds (2, 6, and 7) were characterized by Fourier-transform infrared and nuclear magnetic resonance spectroscopy, as well as mass spectrometry and elemental analysis. The photophysical properties of the BODIPY-substituted cyclotriphosphazenes (6 and 7) were investigated by UV-Vis and fluorescence emission spectroscopy in water and water/solvent mixtures. It was found that the target compounds were soluble in water and could be potential candidates as water-soluble fluorescent dyes for the desired applications.

## 1. Introduction

Water-soluble fluorescent compounds are generally used to define and label targets in aqueous systems [1]. Compared to other fluorescent dyes, boron-dipyrromethene (BODIPY) dyes have special features, like strong absorption in the visible region, narrow emission bands, high quantum yields, and high stability [2–5]. BODIPY dyes mainly have good solubility in organic solvents, but not in water [6]. On the other hand, a great deal of applications, such as fluorescence imaging and metal ion detection, are done in aqueous media and are frequently inhibited by the weak solubility of these dyes in water [7–11]. In general, there are 2 options to overcome this problem: one is linkage of water-soluble groups to the BODIPY core [12] and the other is linkage of the BODIPY dyes to a water-soluble compound [13,14].

Recently, there has been substantial interest in cyclophosphazenes because they not only have an extensive stability range but they can also ensure excellent photophysical properties in combination with suitable fluorescent dyes, such as BODIPYs [15–19] and pyrene derivatives [20–22]. Hexachlorocyclotriphosphazene is a versatile starting scaffold for the synthesis of new compounds, as the chlorine groups attached to the phosphorus atoms are easily substituted by various nucleophiles to form reactive cyclotriphosphazenes. According to the literature, phosphazene compounds generally have good solubility in organic solvents. In order to make these compounds water-soluble, hydrophilic groups should be present in the molecular structure. There are several examples of water-soluble phosphazenes in the literature, but to the best of our knowledge, most of them are focused on polyphosphazenes [23–28]. Thus, the design and synthesis of water-soluble cyclotriphosphazene compounds is particularly important for studies in aqueous media.

**Figure 1:**
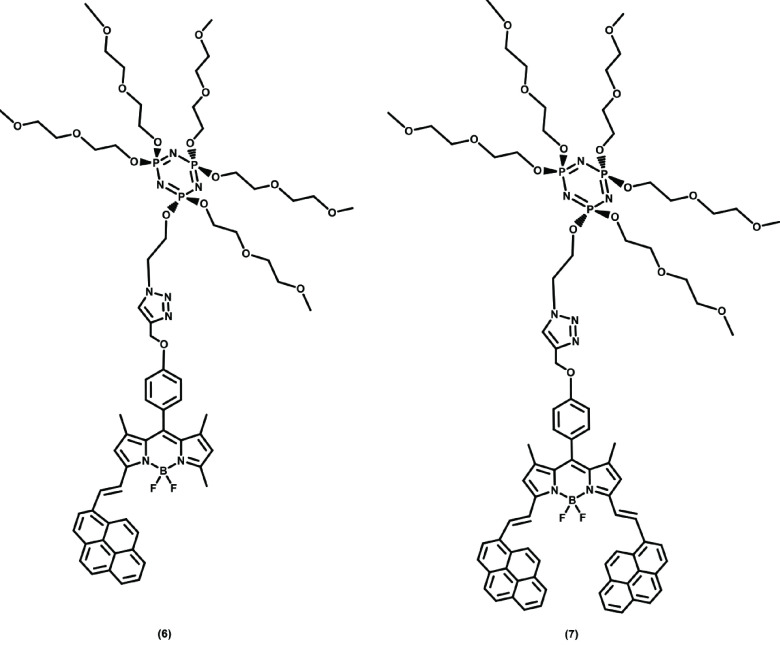
Chemical structures of novel cyclotriphosphazene compounds 6 and 7.

**Figure 2:**
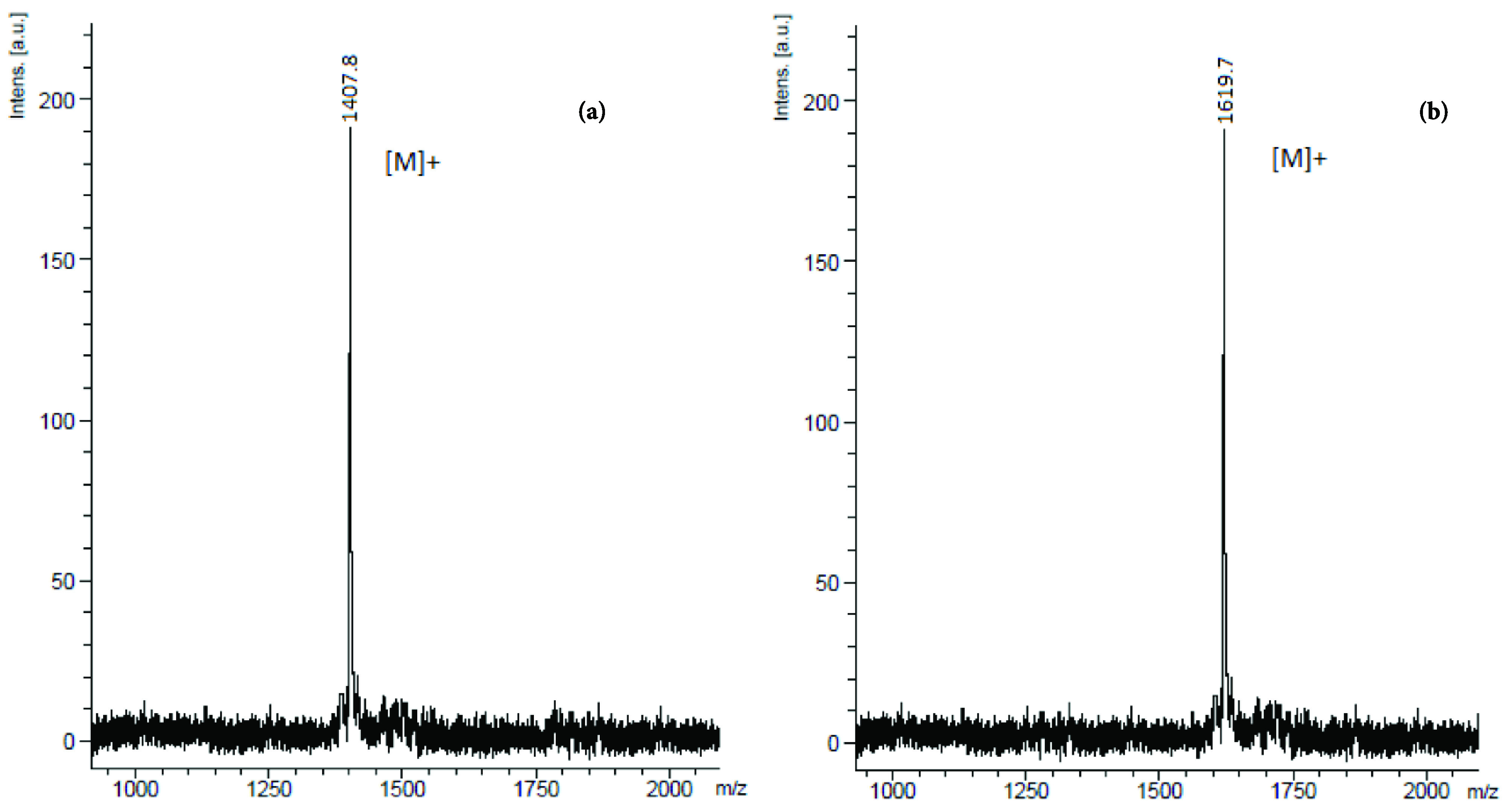
MALDI TOF-MS spectra of compounds (a) 6 and (b) 7. MALDI matrix: 1,8,9-anthracenetriol (20 mg/mL THF).

**Figure 3:**
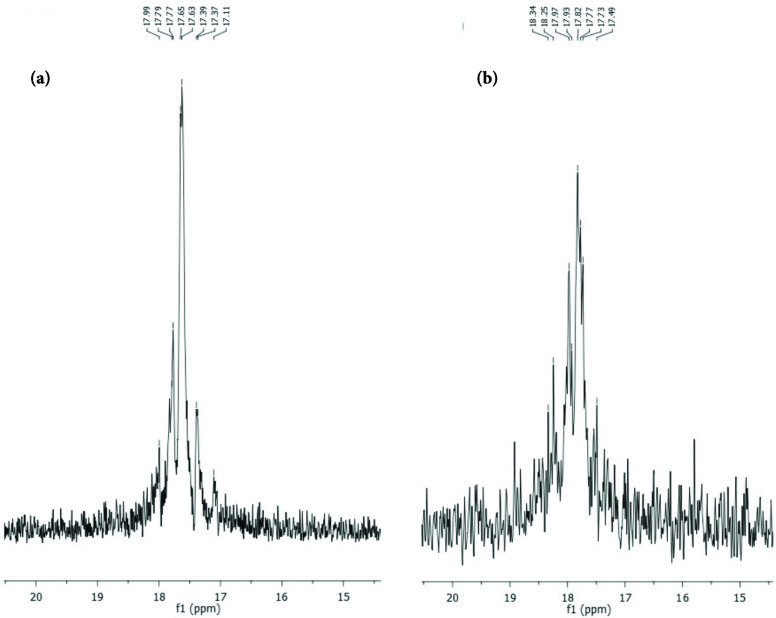
^31^ P NMR spectra of (a) compound 6 and (b) compound 7.

This study aimed at the synthesis of water-soluble novel cyclotriphosphazene compounds (6 and 7) with fluorescent properties. For this purpose, mono- and distyryl pyrene-substituted BODIPYs (4 and 5) were used to introduce fluorescent properties to the cyclotriphosphazene core and diethylene glycol methylether (DEGME) was used to make these compounds water-soluble. Novel cyclotriphosphazene compounds (6 and 7) (Figure 1) were defined using EA, FTIR, MS (Figure 2), and ^1^ H, ^13^ C, and ^31^ P NMR (Figure 3) spectroscopy. Photophysical properties of these cyclotriphosphazenes were investigated by UV-Vis and fluorescence spectroscopy and compared with their precursor BODIPYs in water and different water-miscible solvents.

## 2. Results and discussion

### 2.1. Synthesis and structural characterization of the compounds

Water-soluble novel cyclotriphosphazene compounds 6 and 7 were prepared according to the Scheme. First, hexachlorocyclotriphosphazene was allowed to react with DEGME at a ratio of 1:5 and compound 1 was obtained. Next, compound 2 was prepared by treating 2-azido-1-ethanol with compound 1. Pyrene-substituted BODIPY compounds 4 and 5 were also prepared according to the Scheme. Finally, cyclotriphosphazene compound 2 underwent click reactions with BODIPYs (4 and 5) in the presence of CuBr and pentamethyldiethylenetriamine (PMDETA), and novel cyclotriphosphazene compounds 6 and 7 were obtained, as shown in Figure 1. Column chromatography was used for purification of the products. The synthesized compounds were characterized by EA, FTIR, MS, and ^1^ H, ^13^ C, and ^31^ P NMR spectroscopy. Mass spectra of compounds 6 and 7 are given in Figure 2. In both spectra, molecular ion peaks were related to the calculated values. ^31^ P NMR spectra of compounds 6 and 7 were shown as the AB2 spin system, owing to the different environments of the 2 different phosphorus atoms on the cyclotriphosphazene ring (Figure 3). However, it was not possible to calculate the coupling constants due to the complexity of the spectra.

**Scheme 1:**
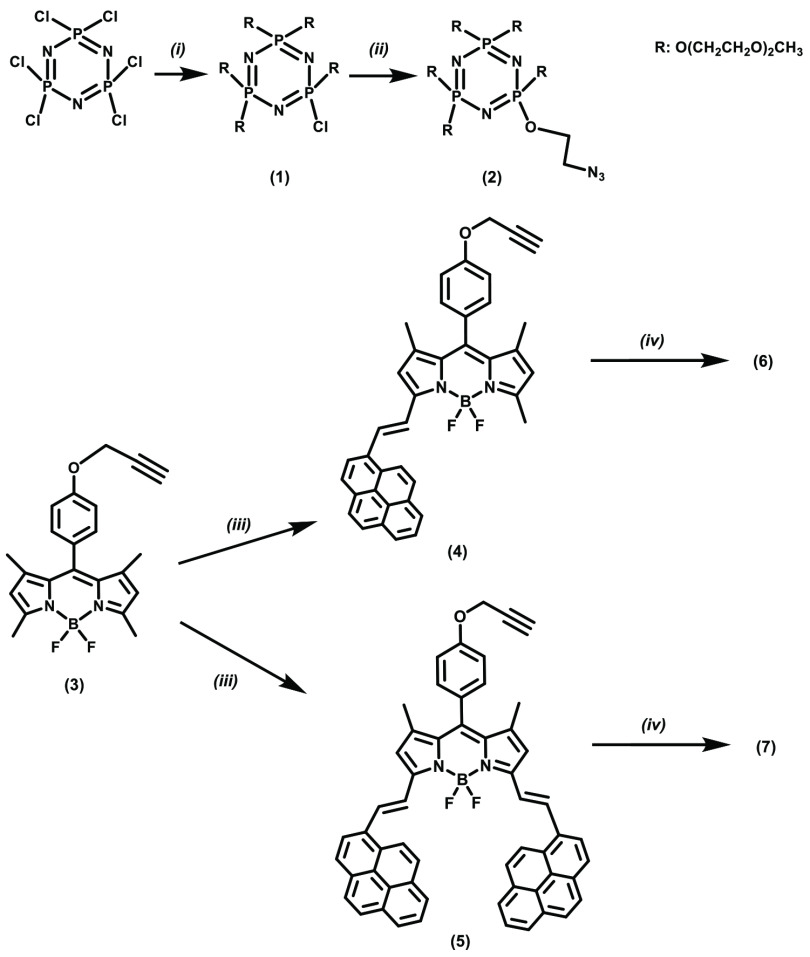
Synthetic pathways to compounds 1–7: (i) HO(CH2 CH2 O)2 CH _3_ , NaH, THF; (ii) 2-azido-1-ethanol, NaH, THF; (iii) 1-pyrenecarboxaldehyde, hexahydropyridine, p-TsOH, methylbenzene; (iv) compound 2, CuBr, PMDETA, DCM.

### 2.2. Photophysical properties of compounds

**Figure 4:**
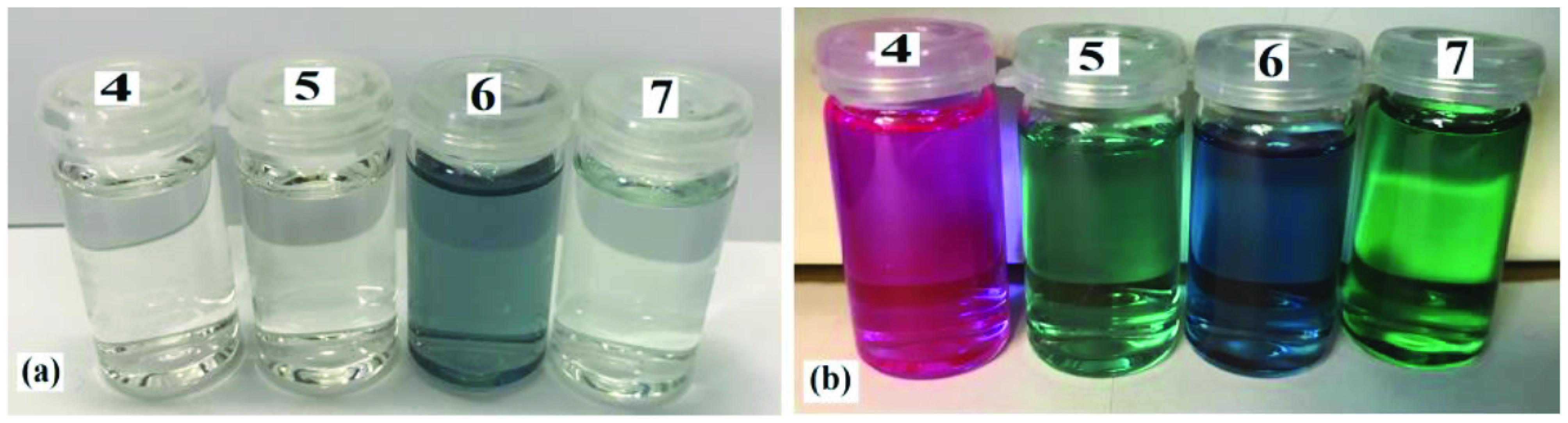
Solubility of compounds (a) in water and (b) in THF.

**Figure 5:**
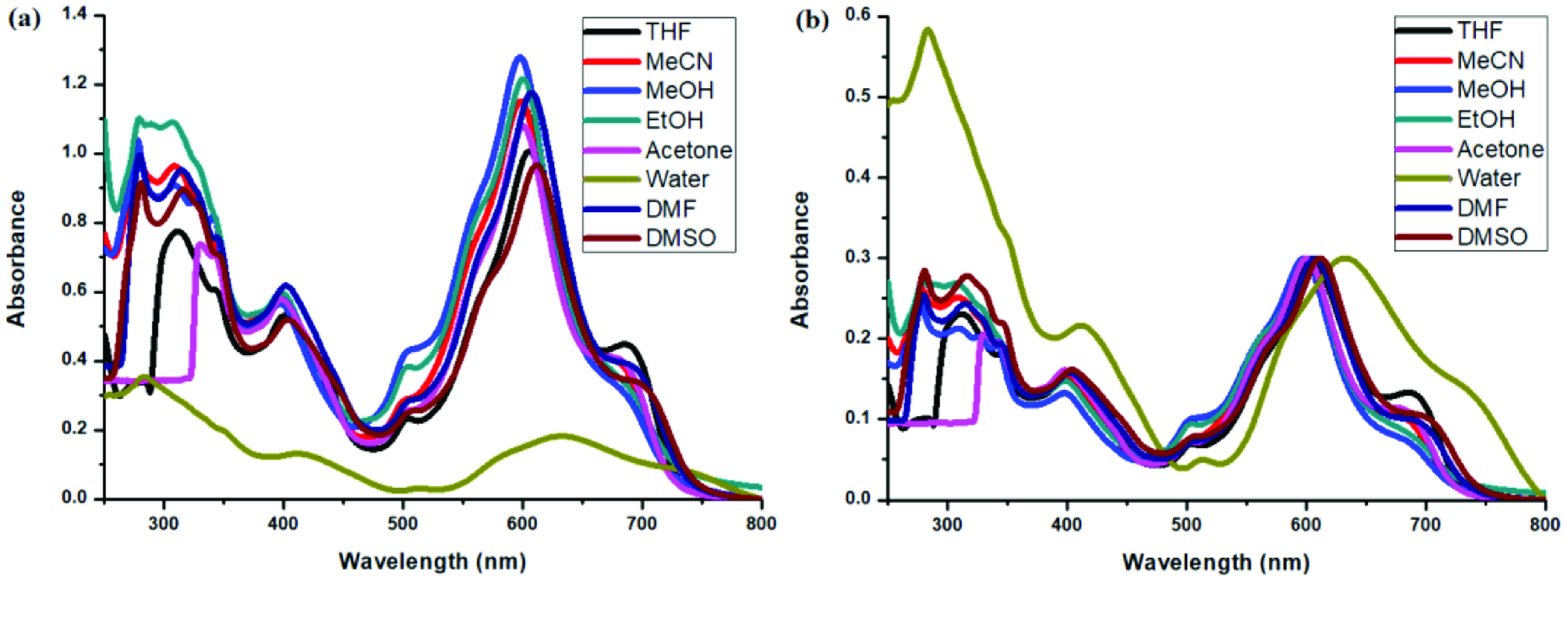
a) UV-Vis absorption spectra of 1 × 10 ^-7^ M compound 6 in different solvents and b) normalized absorption spectra of compound 6 in different solvents.

**Figure 6:**
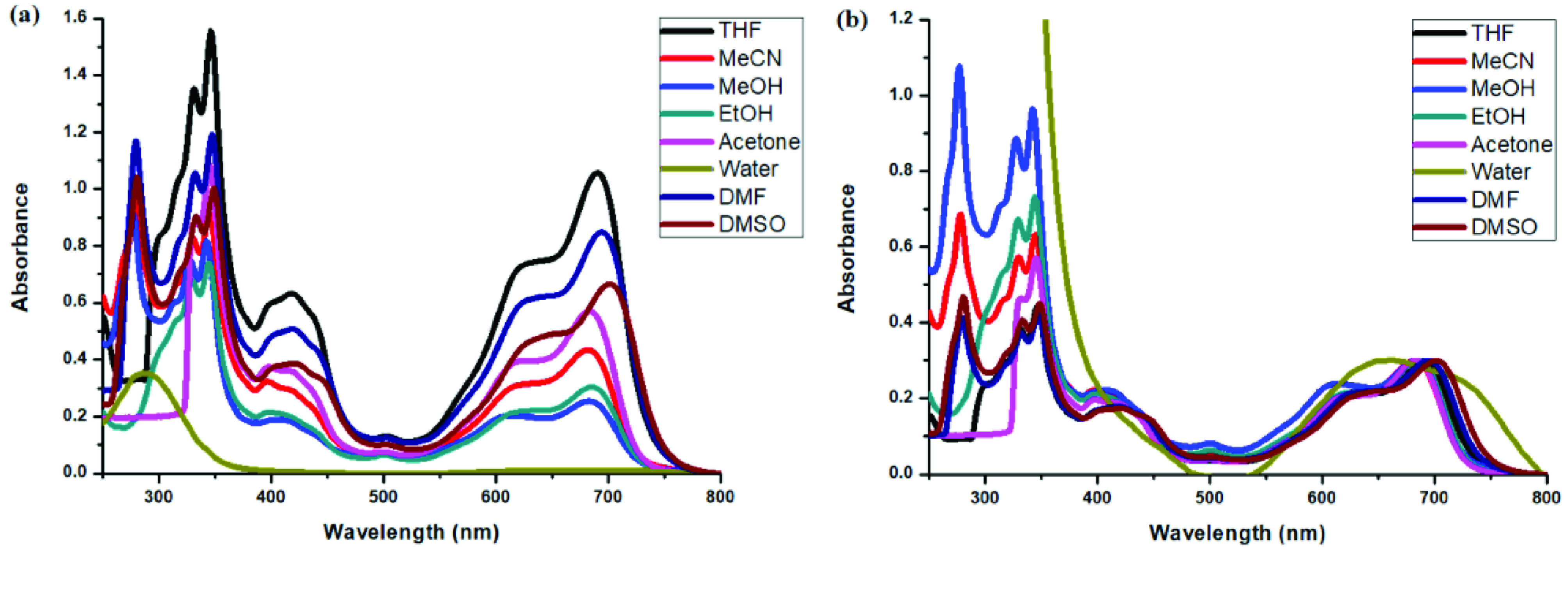
a) UV-Vis absorption spectra of 1 × 10 ^-7^ M compound 7 in different solvents and b) normalized absorption spectra of compound 7 in different solvents.

**Figure 7:**
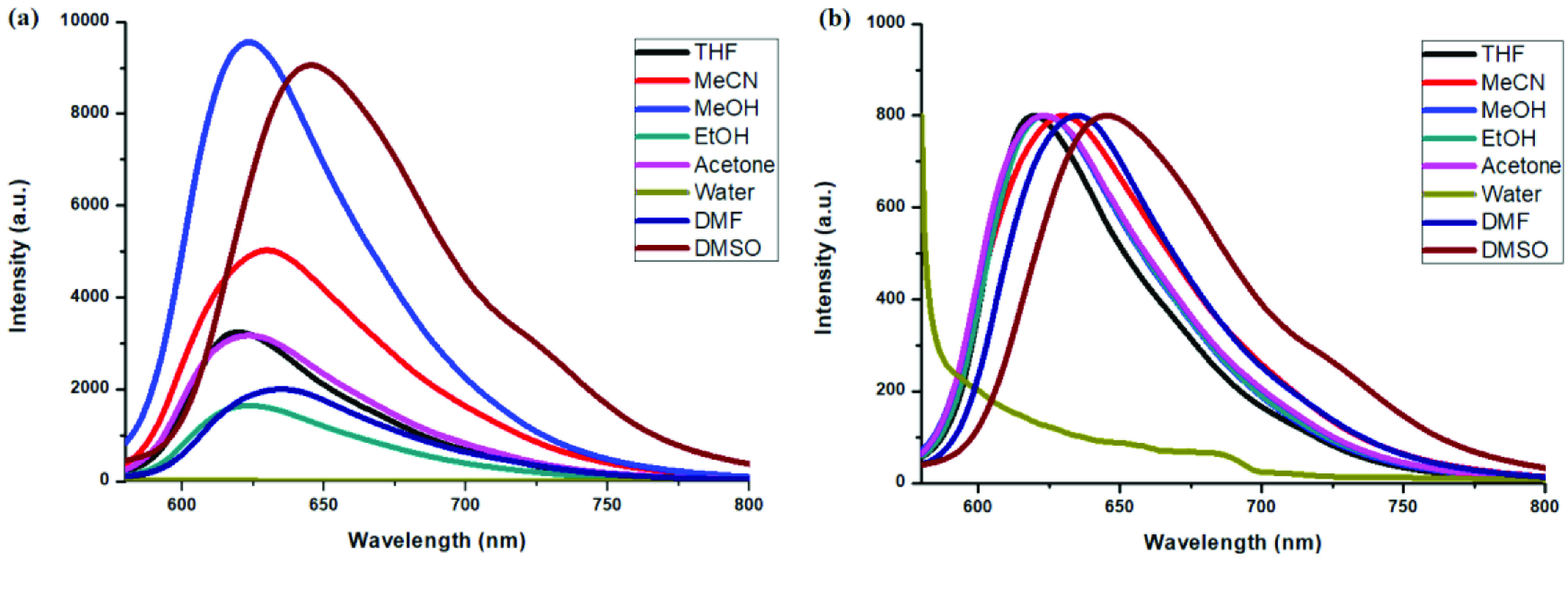
a) Emission spectra of compound 6 in different solvents and b) normalized emission spectra of compound 6 in different solvents (λex = 570 nm).

**Figure 8:**
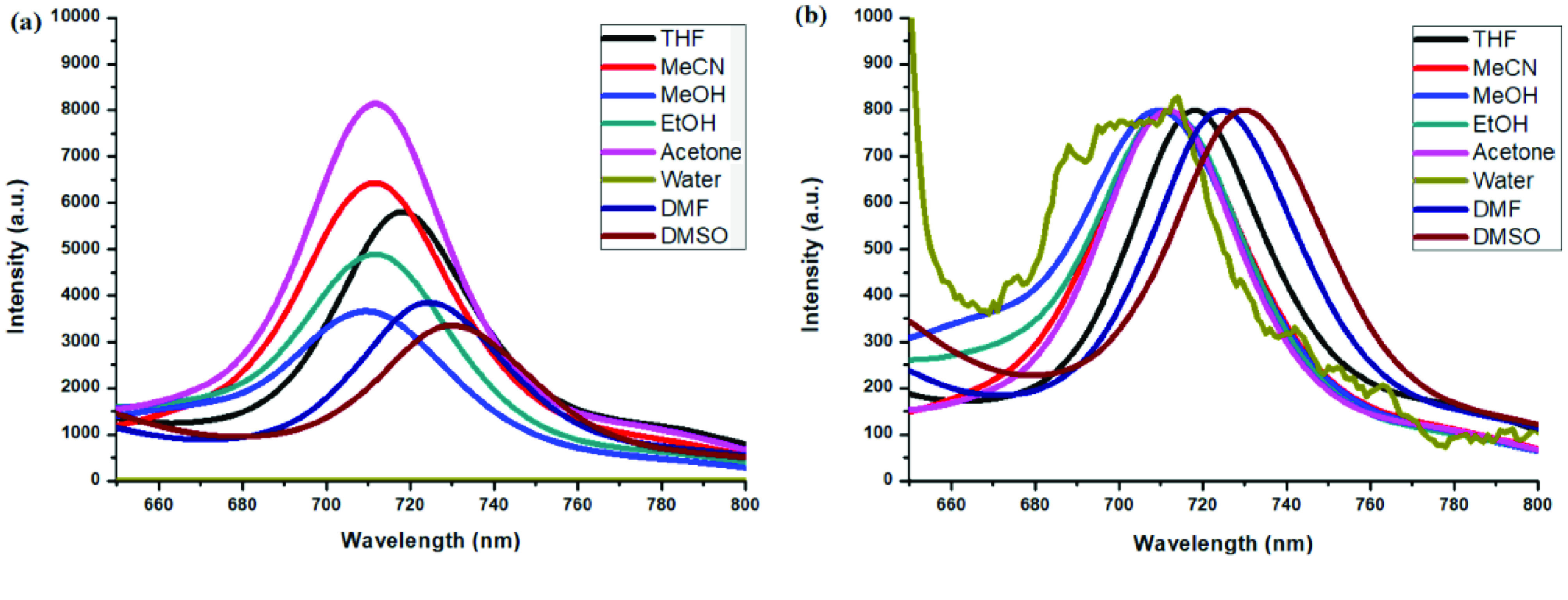
a) Emission spectra of compound 7 in different solvents and b) normalized emission spectra of compound 7 in different solvents (λex = 640 nm).

**Figure 9:**
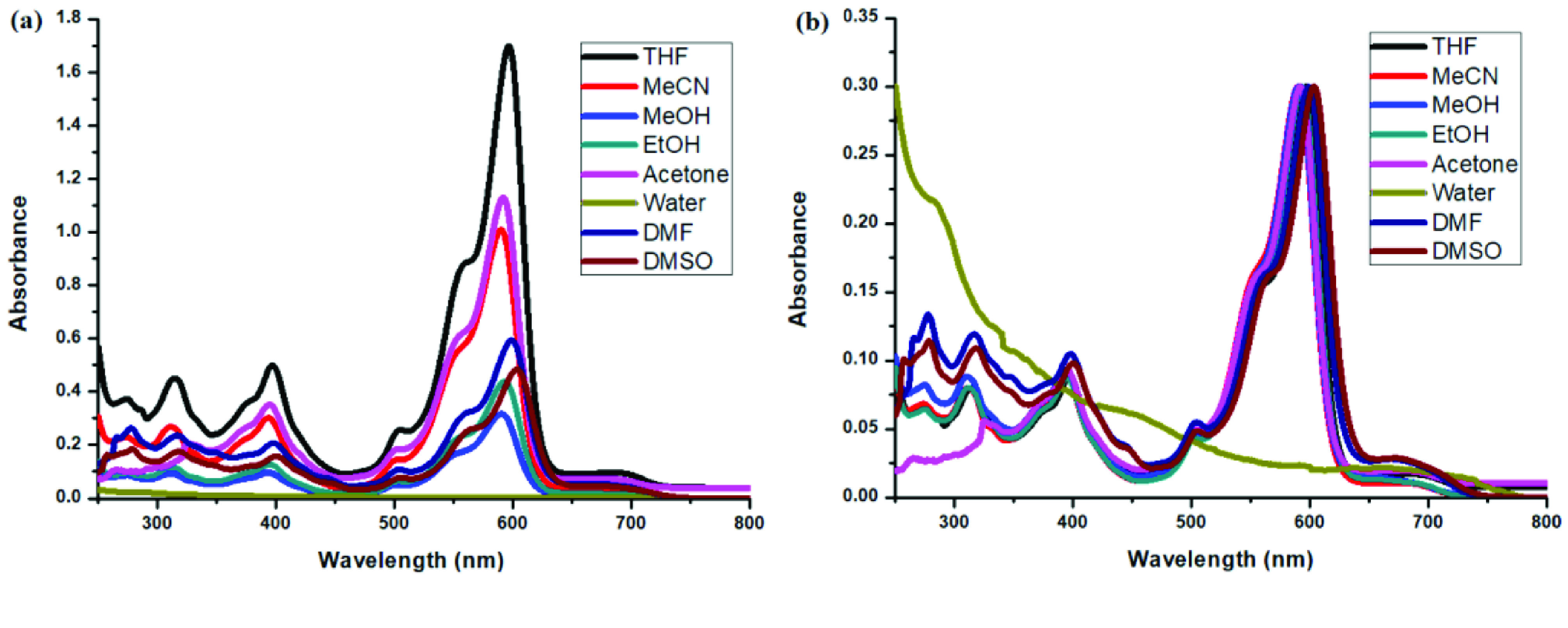
a) UV-Vis absorption spectra of 1 × 10 ^-7^ M compound 4 in different solvents and b) normalized absorption spectra of compound 4 in different solvents.

**Figure 10:**
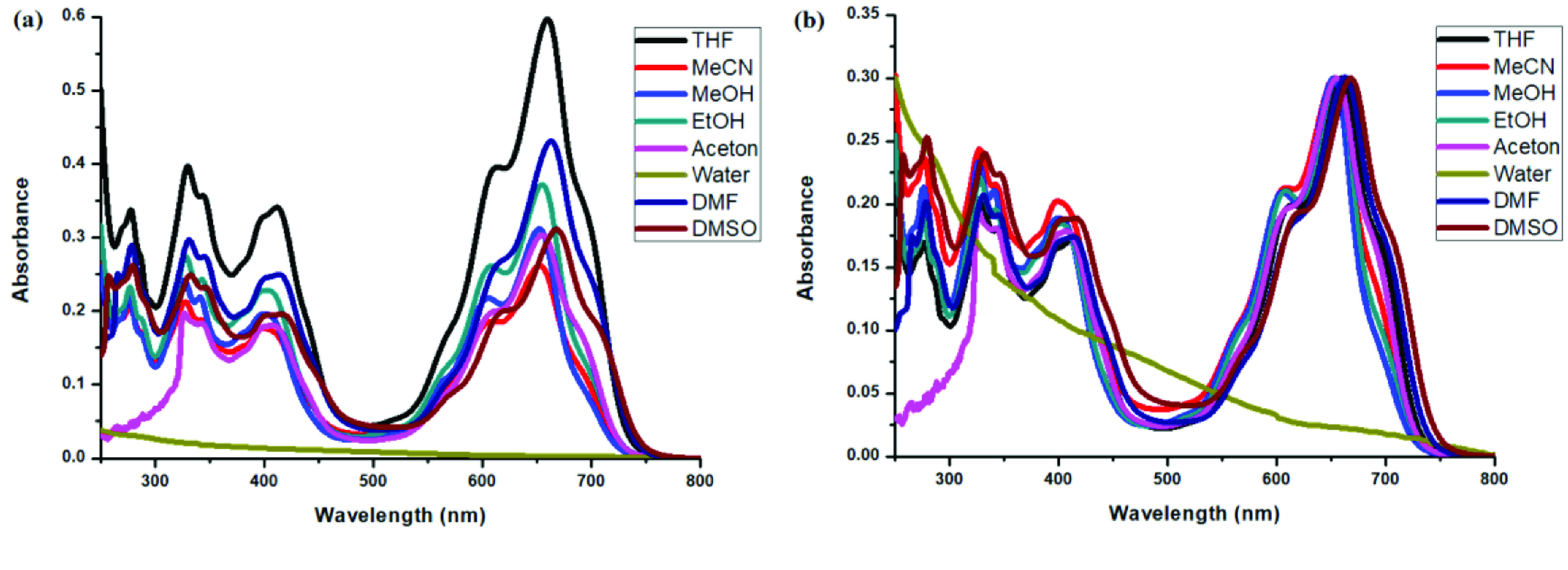
a) UV-Vis absorption spectra of 1 × 10 ^-7^ M compound 5 in different solvents and b) normalized absorption spectra of compound 5 in different solvents.

**Figure 11:**
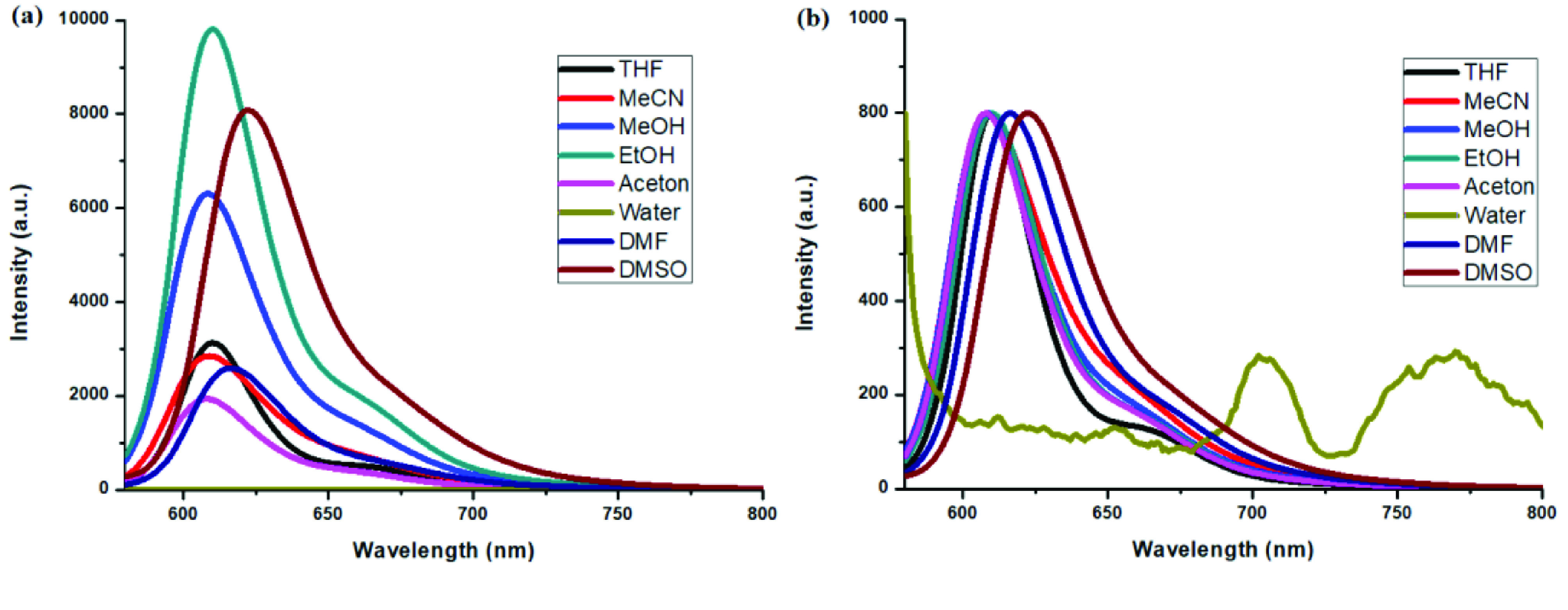
a) Emission spectra of compound 4 in different solvents and b) normalized emission spectra of compound 4 in different solvents (λex = 570 nm).

**Figure 12:**
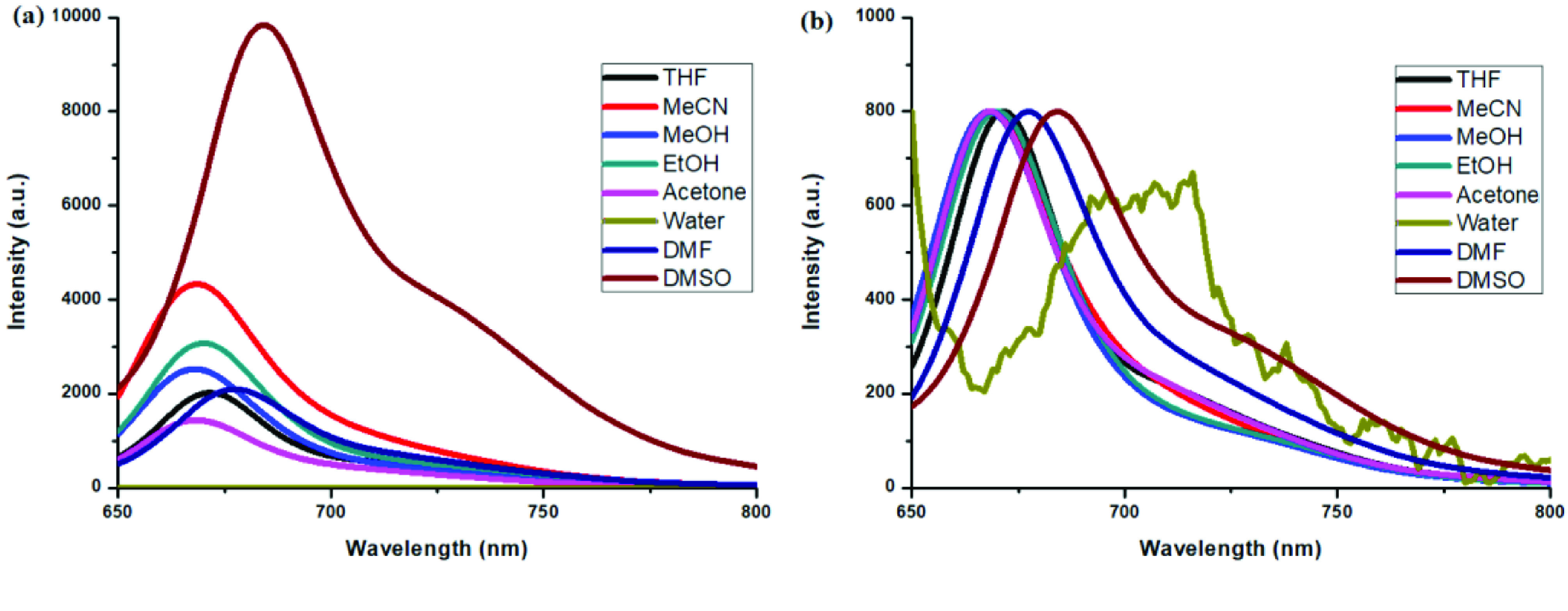
a) Emission spectra of compound 5 in different solvents and b) normalized emission spectra of compound 5 in different solvents (λex = 640 nm).

**Figure 13:**
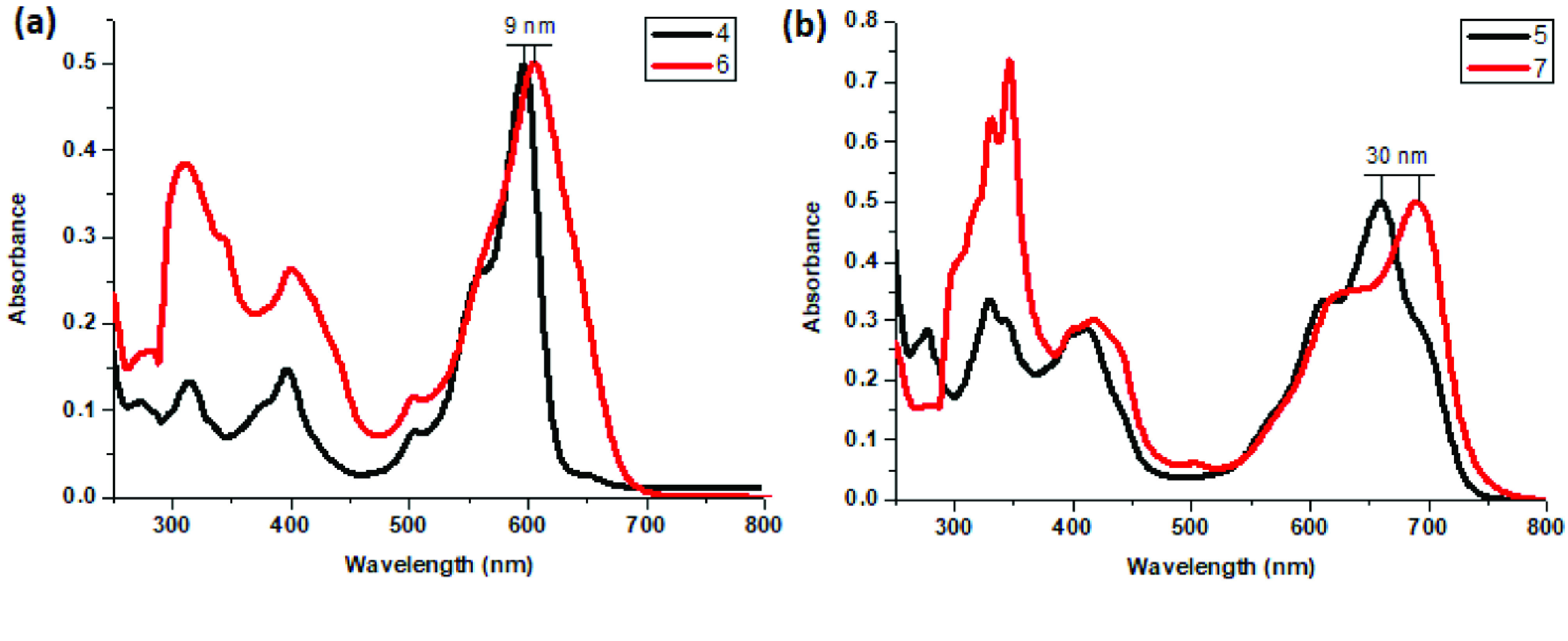
a) Normalized absorbance spectra of compounds 4 and 6 in THF and b) normalized absorbance spectra of compounds 5 and 7 in THF.

**Figure 14:**
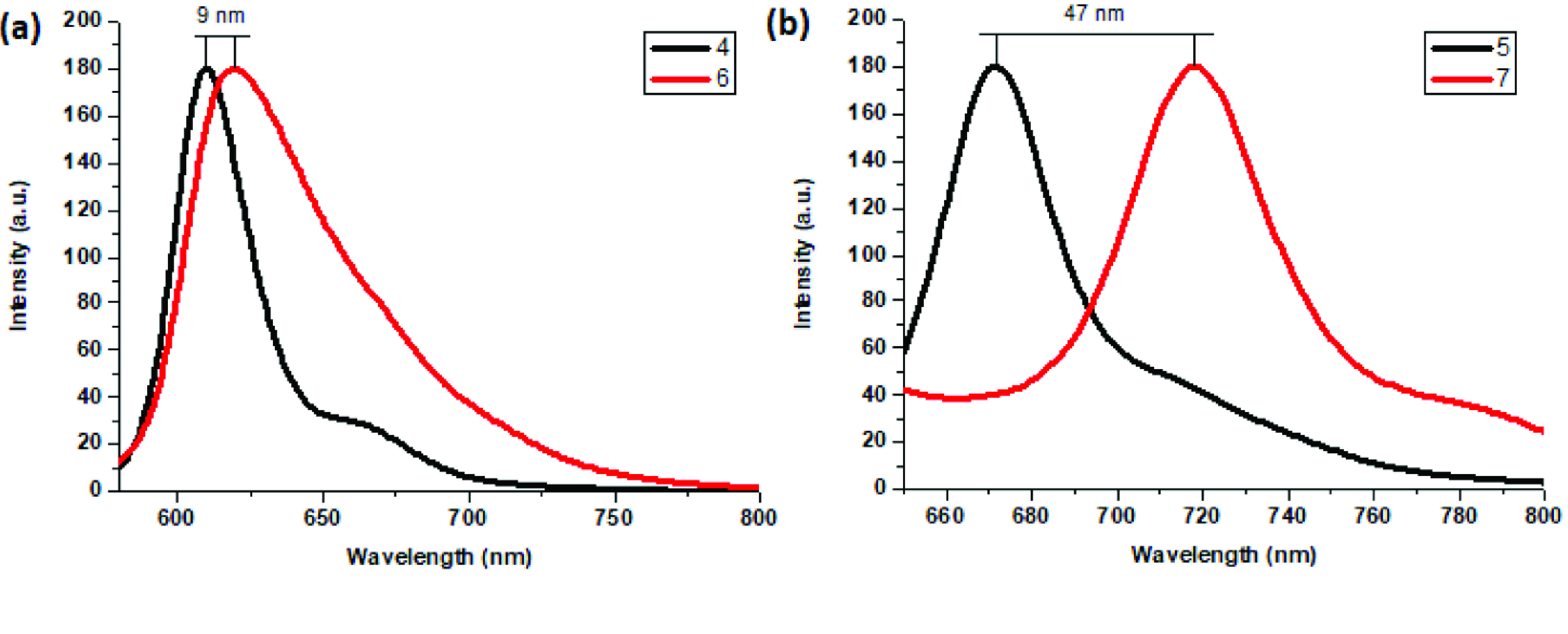
a) Normalized emission spectra of compounds 4 and 6 in THF (λex = 570 nm) and b) normalized emission spectra of compounds 5 and 7 in THF (λex = 640 nm).

Photophysical properties of compounds 6 and 7 and their counterpart BODIPYs 4 and 5 were investigated using UV-Vis and fluorescence spectroscopy. Spectroscopic evaluation of the compounds was performed in water and different water-miscible solvents, such as methanol (MeOH), ethanol (EtOH), acetone, tetrahydrofuran (THF), acetonitrile (MeCN), dimethylformamide (DMF), and dimethyl sulfoxide (DMSO). BODIPY compounds 4 and 5 were not soluble in water, but DEGME and pyrene-BODIPY-substituted novel cyclotriphosphazene compounds 6 and 7 were soluble in water. The solubility of compounds 4–7 in water and in THF is given in Figures 4a and 4b, respectively. The absorption and normalized absorption spectra of compounds 6 and 7 in water and watermiscible solvents are given in Figures 5 and 6. The emission and normalized emission spectra of compounds 6 and 7 in water and water-miscible solvents are given in Figures 7 and 8. The absorption maxima of compound 6 varied between 598 and 611 nm (Figure 5) and the absorption maxima of compound 7 varied between 682 and 701 nm (Figure 6) in different solvents. The fluorescence emission maxima of compound 6 varied between 619 and 645 nm (Figure 7) and the fluorescence emission maxima of compound 7 varied between 709 and 730 nm (Figure 8) in different solvents. The variations in the absorption and emission maxima values in different solvents were related to the solvent systems used, as they can significantly affect the optical properties of the dye molecule due to changing polarities [29]. However, the fluorescence emission spectra obtained in more polar solvents (water, MeOH, EtOH, MeCN) showed negative solvatochromism. This negative solvatochromism has been observed in previously reported pyrenyl systems and is probably due to the disruption of the π-stacking interactions in more polar solvents [30]. In addition, the absorption and emission spectra of the precursor BODIPYs (4 and 5) in water and water-miscible solvents are demonstrated in Figures 9–12. The absorption and fluorescence spectra of 6 and 7 were similar to the pyrene-substituted BODIPY precursors, since the DEGME moiety did not have any optical properties in the UV-Vis region. It is well known that cyclotriphosphazenes are optically transparent in the UV-Vis region and their photophysical properties can be adjusted according to an attached moiety [31,32]. Absorptions and emissions of compounds 6 and 7 and their precursor BODIPYs (4 and 5) were investigated in THF, which is a good solvent for all compounds, at a concentration of 1 × 10 ^-6^ M. As can be seen in Figures 13 and 14, the difference between the absorption and emission maxima of compounds 4 and 6 was 9 nm. However, the difference between the absorption maxima of compounds 5 and 7 was 30 nm, and the difference between the emission maxima of compounds 5 and 7 was 47 nm. These bathochromic shifts were presumably due to π–π interactions of the pyrene components that were more effective on distyryl (5 and 7) than monostyryl (4 and 6) compounds because of the increased number of pyrene components. The fluorescence quantum yields (ΦF) of compounds 4–7 in THF were predicted by comparison with rhodamine 6G in water (ΦF = 0.95) [33] and ZnPC in DMSO (ΦF = 0.18) [34] as standards for BODIPY derivatives. Fluorescence lifetimes were directly measured and monoexponential calculations were used for all compounds (4–7). The photophysical properties, lifetimes (τ F), and Φf values of compounds 4–7 in THF are summarized in the Table.

**Table:** Photophysical properties of the target compounds.

Compound	λabs (nm)	λems (nm)	ε (THF, L mol−1 cm ^-1^ )	τ F (ns)	Φf
4	396, 596	610	41240	3.615 ± 0.004	0.50
5	412, 659	671	66580	1.194 ± 0.020	0.21
6	400, 605	619	49920	3.650 ± 0.005	0.53
7	418, 690	718	69850	1.349 ± 0.003	0.25

### 2.3. Conclusion

In the current study, 2 novel cyclotriphosphazene compounds (6 and 7) were synthesized using click reactions and characterized by FTIR, MS, ^1^ H, ^13^ C, and ^31^ P NMR spectroscopies and EA. The photophysical properties of these compounds (6 and 7) were investigated by UV-Vis and fluorescence spectroscopy and compared with their corresponding BODIPY (4 and 5) moieties. The fluorescence quantum yield (ΦF) values of novel cyclotriphosphazenes 6 and 7 were determined and their fluorescence lifetimes were directly measured. These novel cyclotriphosphazene compounds had solubility in water and good solubility in water-miscible solvents.

## 3. Experimental

### 3.1. Materials and methods

Thin-layer chromatography (TLC) was carried out on silica gel plates with F254 indicator. Purification of the compounds was carried out on silica gel-filled columns with defined eluents. Deuterated chloroform (CDCl _3_ ) and the subsequent chemicals were purchased from Merck (Darmstadt, Germany): p-hydroxybenzaldehyde, N,Ndiethylethanamine, boron trifluoride-ethyl etherate, hexahydropyridine, potassium carbonate, sodium azide, sodium sulfate (Na _2_ SO _4_ ) , sodium hydride (NaH), PMDETA, glacial acetic acid, silica gel 60, methylene chloride (DCM), ethyl acetate, n-hexane, methylbenzene, acetone, THF, MeCN, MeOH, EtOH, DMSO, and DMF. Hexachlorocyclotriphosphazene, p-toluene sulfonic acid (p-TsOH), 2,4-dimethyl pyrrole, trifluoroacetic acid, 2,3-dichloro-5,6-dicyano-1,4-benzoquinone, DEGME, magnesium perchlorate, and 1,8,9-anthracenetriol were purchased from Sigma-Aldrich (St. Louis, MO, USA). 1-Pyrene carboxaldehyde, 2-bromo ethanol, CuBr, and 3-bromo-1-propyne were purchased from Alfa-Aesar (Haverhill, MA, USA). A Thermo Finnigan Flash 1112 instrument (Waltham, MA, USA) was used for EA. A Shimadzu 2001 UV spectrophotometer (Kyoto, Japan) was used to record the UV/Vis spectra and a Varian Eclipse spectrofluorometer (Palo Alto, CA, USA) was used to record fluorescence emission spectra. The fluorescence lifetime measurements were done using a Horiba-Jobin-Yvon-SPEX Fluorolog 3-2iHR instrument (Kyoto, Japan). Signal receiving was carried out with a TCSPC modulus. A PerkinElmer Spectrum 100 FTIR spectrophotometer (Waltham, MA, USA) was used for IR measurements. A Bruker Daltonics Microflex mass spectrometer (Billerica, MA, USA) was used for recording mass spectra. The ^1^ H, ^13^ C, and ^31^ P NMR spectra were recorded in CDCl _3_ solution on a Varian 500 MHz spectrometer.

### 3.2. Synthesis

2-Azido-1-ethanol [35], BODIPY 3 [36], and trimeric compound 1 (P _3_ N _3_ -(O-CH _2_ -CH _2_ -O-CH _2_ -CH _2_ -OCH _3_ ) _5_ Cl) [37] were prepared and purified according to the literature procedures.

#### 3.2.1. Synthesis of compound 2

2-Azido-1-ethanol (84 mg, 1.5 mmol) in 10 mL of THF was put into a 2-necked round-bottomed flask and cooled in an ice bath under Ar atm. NaH (46 mg, 3 mmol) in 20 mL of THF was added to the flask slowly. Finally, trimeric compound 1 (500 mg, 1 mmol) in 20 mL of THF was added slowly and stirred for 24 h at room temperature (rt). The reaction mixture was filtered and the solvent was evaporated using a rotary evaporator. Compound 2 (520 mg, 0.64 mmol) was purified by silica gel column chromatography [eluent: n-hexane: THF (3:2)] in 89% yield. Anal. calcd. for C _27_ H _59_ P _3_ N _6_ N _6_ : C, 39.71%; H, 7.28%, N, 10.29%; found: C, 39.91%, H, 7.68%, N, 10.23%. MALDI-MS (m/z): 816.9 [M] ^+^ found; 816.7 m/z calcd. FTIR ν max/cm ^-1^ : 2917, 2874 (-CH str), 2111 (-N _3_ ) , 1228 (-P=N-), 1061, 979 (-P-O-C str). ^1^ H NMR (CDCl _3_ , δ : ppm) 1.74 (t, 2H, -CH _2_ -N _3_ ) , 3.28 (s, 15H, -O-CH _3_ -); 3.44–3.62 (m, 42H, -O-CH _2_ ) . ^13^ C NMR (CDCl _3_ , δ : ppm) 72.5, 71.8, 70.1, 61.4, 58.7, 53.4. ^31^ P NMR (CDCl _3_ , δ : ppm) 17.76 (broad singlet).

#### 3.2.2. Synthesis of compound 4

A mixture of compound 3 (200 mg; 0.5 mmol), 1-pyrene carboxaldehyde (122 mg; 0.6 mmol), p-TsOH (143 mg; 0.8 mmol), and hexahydropyridine (0.1 mL) was boiled under reflux condenser in toluene (50 mL). A Dean-Stark apparatus was used to remove water formed in the reaction. After solvent evaporation, the crude product was purified on silica gel with an EtOAc:n-hexane (1:2) solvent system. Purple-colored compound 4 was collected (190 mg, 41% yield). Anal. calc. for C _39_ H _29_ BF _2_ N _2_ O: C, 79.33%; H, 4.95%; N, 4.74%; found: C, 78.25%; H, 4.50%; N, 4.70%. MALDI-MS (m/z): 592.3 [M] ^+^ and 571.7 [M-F] ^+^ found; 591.5 m/z calcd. FTIR ν max/cm ^-1^ : 3292 (-C≡C-H), 3040 (Ar-C-H), 2955–2859 (-C-H), 1591 (-C=N-), 1483 (B-N), 1383 (B-F). ^1^ H NMR (THF-d8 , δ : ppm) 8.90–8.10 (m, 9H, pyrene-H), 7.37 (d, 2H, J = 7.7, Ar-H), 7.23 (d, 2H, J = 7.7, Ar-H), 7.11 (d, 1H, J = 16.1, HC=C-H), 6.12 (d, 1H, J = 16.1, H-C=C-H), 5.54 (s, 2H, pyrrole-H), 4.86 (d, 2H, J = 2.4, -CH _2_ ), 3.08 (s, 1H, -C≡CH), 2.61 (s, 3H, CH _3_ ), 1.63 (s, 3H, CH _3_ ), 1.33 (s, 3H, CH _3_ ). ^13^ C NMR (CDCl _3_ , δ : ppm) 177.9, 176.9, 161.3, 110.7, 110.0, 109.9, 107.9, 107.5, 107.3, 107.2, 106.3, 102.9, 101.5, 100.8, 99.0, 98.3, 98.0, 68.5, 67.2, 33.1, 27.7, 23.9, 22.1.

#### 3.2.3. Synthesis of compound 5

According to the above procedure, compound 3 (200 mg; 0.5 mmol), 1-pyrene carboxaldehyde (276 mg; 1.2 mmol) p-TsOH (342 mg; 1.8 mmol), and hexahydropyridine (0.2 mL) were boiled under reflux condenser in toluene (80 mL). A Dean-Stark apparatus was used to remove water formed in the reaction. After solvent evaporation, the crude product was purified on silica gel using an EtOAc:n-hexane (1:2) solvent system. Greencolored compound 5 was collected (180 mg, 22% yield). Anal. calc. for C _56_ H _37_ BF _2_ N _2_ O: C, 83.79%; H, 4.65%; N, 3.49%; found: C, 82.25%; H, 4.60%; N, 3.47%. MALDI-MS (m/z): 804.1 [M] ^+^ and 783.0 [M-F] ^+^ found; 804.7 m/z calcd. FTIR ν max/cm ^-1^ : 3262 (-C≡C-H), 3037 (Ar-C-H), 2964–2922 (-C-H), 1589 (-C=N-), 1475 (B-N), 1385 (B-F). ^1^ H NMR (DMSO-d6, δ : ppm) 8.90–7.90 (m, 18H, pyrene-H), 7.49 (d, 2H, J = 7.7, Ar-H), 7.25 (d, 2H, J = 7.7, Ar-H), 7.12 (d, 2H, J = 16.2, HC=C-H), 6.13 (d, 2H, J = 16.2, H-C=C-H), 5.62 (s, 2H, pyrrole-H), 4.94 (d, 2H, J = 2.4, -CH _2_ ) , 3.66 (s, 1H, -C≡CH), 1.59 (s, 6H, CH _3_ ) . ^13^ C NMR (CDCl _3_ , δ : ppm) 177.9, 176.9, 161.3, 110.7, 110.0, 109.9, 107.9, 107.5, 107.3, 107.2, 106.3, 102.9, 101.5, 100.8, 99.0, 98.3, 98.0, 68.5, 67.2, 33.1, 27.7, 23.9, 22.1.

#### 3.2.4. Synthesis of compound 6

A mixture of trimeric compound 2 (200 mg; 0.3 mmol), compound 4 (177 mg; 0.3 mmol), CuBr (72 mg; 0.5 mmol), and PMDETA (88 mg; 0.50 mmol) in dry DCM (50 mL) was stirred in a flask for 48 h at rt under Ar atm. Next, it was poured into water and extracted with DCM. The extract was washed with water and dried over anhydrous Na _2_ SO _4_ . The remaining product was purified by silica gel column chromatography [eluent: n-hexane: THF (3:2)] and compound 6 was obtained as a dark-purple fraction (160 mg, 30%). Anal. calc. for C _66_ H _88_ BF _2_ N _8_ O _17_ P _3_ : C, 56.33; H, 6.30; N, 7.96%; found: C, 55.31%; H, 6.60%; N, 7.23%. MALDI-MS (m/z): 1407.8 [M] ^+^ found; 1407.2 m/z calcd. FTIR ν max/cm ^-1^ : 2920, 2866 (-CH str), 1654 (-C=N-), 1602 (-C=C-), 1542 (-N=N-), 1463 (-N-N-), 1396 (B-N), 1305 (B-F), 1225 (-P-N-P-), 1275 (-C-N-), 1260 (P=N str), 1139 (-P- O-), 1106 (C-O-C str), 1039, 975 (P-O-C str). ^1^ H NMR (CDCl _3_ , δ : ppm) 1.43 (s, 3H, -CH _3_ ) , 1.45 (s, 3H, -CH _3_ ) , 2.56 (s, 3H, -CH _3_ ) , 3.40 (s, 15H, -CH _3_ ) , 3.5–4.0 (m, 44H, CH _2_ ) , 6.0 (s, 1H, pyrrole-H), 6.02 (s, 1H, pyrrole-H), 6.20 (s, 2H, -OCH _2_ -), 6.68 (d, ^3^ JHH = 8.5 Hz, 2H, Ar-H), 6.90 (d, ^3^ JHH = 16.2 Hz, 1H, -HC=CH-), 6.96 (d, ^3^ JHH = 16.2 Hz, 1H, -HC=CH-), 7.10 (d, ^3^ JHH = 8.5 Hz, 2H, Ar-H), 7.91 (s, 1H, -NH), 8.61–8.04 (m, 9H, pyrene-H). ^13^ C NMR (CDCl _3_ , δ : ppm) 158.2, 142.3, 138.8, 136.0, 134.6, 133.4, 132.0, 129.1, 128.6, 128.3, 128.0, 127.9, 126.6, 125.6, 124.1, 123.5, 119.1, 118.8, 115.2, 114.2, 113.4, 111.8, 109.4, 72.3, 71.6, 71.5, 70.1, 59.3, 55.5, 53.5, 52.4, 19.5, 14.3, 14.1. ^31^ P NMR (CDCl _3_ , δ : ppm): 17.9–17.1 (m).

#### 3.2.5. Synthesis of compound 7

According to the above procedure, compound 7 as a dark-green fraction (60 mg; 25%) was obtained using trimeric compound 2 (100 mg; 0.1 mmol), compound 5 (80 mg; 0.1 mmol), CuBr (29 mg; 0.2 mmol), and PMDTA (35 mg; 0.2 mmol) in dry DCM (20 mL). Anal. calc. for C _83_ H _96_ BF _2_ N _8_ O _17_ P _3_ : C, 61.56%; H, 5.38%; N, 6.92%; found: C, 60.45%; H, 5.75%; N, 6.63%. MALDI-MS (m/z): 1619.7 [M] ^+^ found; 1619.5 m/z calcd. FTIR ν max/cm ^-1^ : 2950, 2860 (-CH str), 1656 (-C=N-), 1602 (-C=C-), 1592 (-N=N-), 1453 (-N-N-), 1396 (B-N), 1305 (B-F), 1229 (-P-N-P-), 1275 (-C-N-), 1260 (P=N str), 1134 (-P-O-), 1104 (C-O-C str), 1033, 979 (P-O-C str). ^1^ H NMR (CDCl _3_ , δ : ppm) 1.95 (s, 3H, -CH _3_ ) , 2.29 (s, 3H, -CH _3_ ) , 3.41 (s, 15H, -CH _3_ ) , 3.5–4.0 (m, 44H, -CH _2_ ) , 5.20 (s, 2H, -OCH _2_ -Ar), 6.00 (s, 1H, pyrrole-H), 6.03 (s, 1H, pyrrole-H), 6.39 (d, ^3^ JHH = 16.2 Hz, 2H, -HC=CH-), 6.52 (d, ^3^ JHH = 16.2 Hz, 2H, -HC=CH-), 7.13 (d, ^3^ JHH = 16.2 Hz, 2H, Ar-H), 7.34 (d, ^3^ JHH = 16.2 Hz, 2H, Ar-H), 7.59 (s, 1H, -NH), 8.61–8.04 (m, 18H, pyrene-H). ^13^ C NMR (CDCl _3_ , δ ; ppm) 158.2, 142.3, 141.8, 140.2, 137.0, 136.0, 135.6, 134.6, 132.7, 130.5, 129.7, 128.3, 127.9, 127.5, 126.6, 125.6, 124.5, 122.8, 119.1, 117.2, 114.2, 108.1, 72.3, 71.6, 71.5, 70.1, 59.3, 55.5, 53.5, 52.4, 14.3, 14.2. ^31^ P NMR (CDCl _3_ , δ : ppm): 17.4–18.3 (m).
